# Reply to: “Enhancement of *Aedes aegypti* susceptibility to dengue by *Wolbachia* is not supported”

**DOI:** 10.1038/s41467-020-19831-5

**Published:** 2020-11-30

**Authors:** Caetano Souto-Maior, Jessica G. King, Larissa M. Sartori, Rafael Maciel-de-Freitas, M. Gabriela M. Gomes

**Affiliations:** 1 Laboratory of Systems Genetics, National Heart Lung and Blood Institute, National Institutes of Health, Bethesda, MD USA; 2 Institute of Evolutionary Biology, University of Edinburgh, Edinburgh, UK; 3 Instituto de Matemática e Estatística, Universidade de São Paulo, São Paulo, Brazil; 4 Laboratório de Transmissores de Hematozoários, IOC, Fundação Oswaldo Cruz, Rio de Janeiro, Brazil; 5 Department of Mathematics and Statistics, University of Strathclyde, Glasgow, UK; 6 Centro de Matemática da Universidade do Porto, Porto, Portugal

**Keywords:** Ecological epidemiology, Pathogens

**Replying to** Ant et al. *Nature Communications* 10.1038/s41467-020-19830-6 (2020)

We recently reanalysed previously published datasets (Souto-Maior et al.^1^ and Ferguson et al.^2^). Using mathematical and statistical models based on these data, we showed that *Wolbachia* strain *w*Mel increases mean and variance of *Aedes aegypti* susceptibility to infection by dengue viruses^3^. Ant et al.^4^ claim that concerns with the data and broader analysis make our conclusions misleading. We herein respond to their comments by demonstrating the robustness of our results to different treatments of the data, and expand our arguments for replacing currently adopted methods by those introduced in our paper.

Ant et al.^4^ describe concerns with one of the datasets^1^ used in our analysis. They highlight that a fraction of the mosquitoes assigned as infected by dengue may be false positives, without which the increased mean susceptibility of the *Wolbachia*-carrying group inferred from the dose–response model might not be found, making our reported conclusions appear as an artefact of the analysis. They criticise Souto-Maior et al.^1^ based on the observation that a small number of negative control mosquitoes were classified as positive, a shortcoming of the original experimental study which neither affects the type of analyses conducted therein nor does it influence the conclusions of King et al.^3^, as per modified analysis suggested by Ant et al.^4^ (Fig. 1). Unfortunately, however, the other study whose data we used in our analysis^2^ did not report negative controls, precluding an assessment of whether the same concerns might apply there. We originally gave both studies the same treatment, and reanalysed the two datasets without alternative filtering or pre-processing^3^, while Ant et al.^4^ highlight one of the studies and propose a threshold for false positives based on the mean cycle threshold (Ct) value of five quantifiable negative controls.

**Fig. 1:**
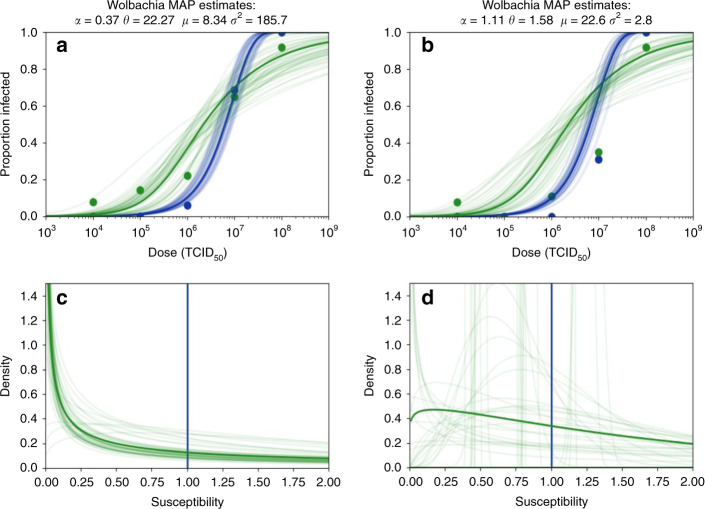
Dose–response curves and associated distributions inferred under different pre-processing of data. Dose–response curves for *Wolbachia*-free (Wolb^−^, in blue) and Wolbachia-carrier (Wolb^+^, in green) populations given data (dots) from Rio de Janeiro, Brazil^1^. The original procedures (King et al.^3^, “Methods”) are replicated in **a** and **c**, showing the bold curves of the maximum a posteriori (MAP) estimate (King et al.^3^, **a**, **c**) and 50 random Monte Carlo Markov Chain estimates, almost all of which have the shape parameter (*α*) smaller than one, giving high densities close to zero susceptibility and a long tail towards larger values (*θ* is the scale parameter). Applying an alternative threshold for false positives based on the mean cycle threshold (Ct) value of five quantifiable negative controls in the original data (**b**, **d**) results in less mosquitoes being classified as infected (noticeable in **b**), and leads to higher estimated mean susceptibility (*μ*) for the *Wolbachia* group and less variance (*σ*^2^). As in King et al.^3^, these analyses exclude the first time point (3 days post infection) as it may represent transient, dose-independent presence of virus rather systemic infection.

To demonstrate how an alternative threshold for false positives applied to Souto-Maior et al.^1^ impacts the results of King et al.^3^, we test sensitivity by fitting the dose–response model to the highlighted dataset with the data pre-processed, as suggested by Ant et al.^4^. The results are shown in Fig. 1, revealing that although estimated parameter values are sensitive to the change in the dataset—as they should be—the qualitative results that *Wolbachia* increases the mean (*μ* > 1) and variance (*σ*^2^ > 0) of mosquito susceptibility to infection by dengue viruses are robust to that change. Quantitatively, the modification proposed by Ant et al.^4^ (plots on the right of the figure) amplifies the increase in mean susceptibility and diminishes the increase in variance, when compared with the original analysis (left). We caution, however, that the suggested pre-processing resulted in a more erratic dose–response trend leading to greater uncertainty.

*Wolbachia*-mediated pathogen blocking in insects is usually characterised by challenging groups of carriers and noncarriers with known pathogen doses and comparing proportions of infected individuals across the two groups to assess susceptibility (illustrated in Fig. 2), and proportions of infected salivary glands to assess infectivity. Most studies (1) decide on a convenient dose for administration of the pathogen and quantify how *Wolbachia* alters the probability of insects becoming infected^5–10^ (Fig. 2c). Others (2) contemplate a range of doses and fit dose–response curves to assess how *Wolbachia* impacts the dose required to infect a certain proportion of insects^2^ (Fig. 2a). Each approach applies only in very special circumstances, which led us to propose a general framework^11,12^ that interpolates between (1) and (2) (Fig. 2b).

**Fig. 2:**
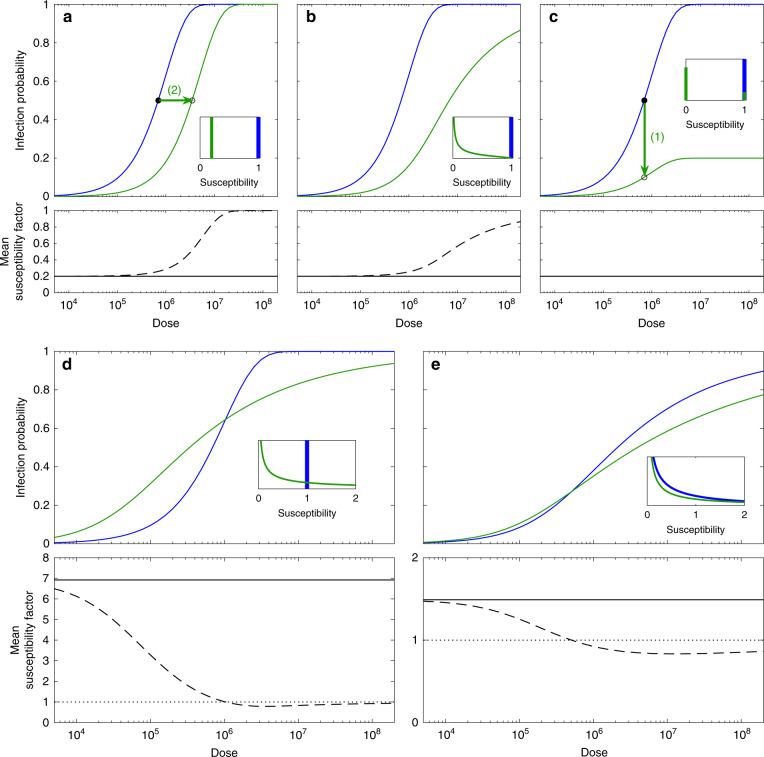
Distributions of susceptibility factors and their impact on dose–response curves. Blue dose–response curves represent infection probabilities in insects without *Wolbachia*, while green represent *Wolbachia* carriers. Insets depict distributions of susceptibility in noncarriers (blue) and carriers (green) normalised such that noncarriers have mean susceptibility one. Solid black lines represent the mean susceptibility of *Wolbachia* carriers, while dashed black curves mimic common procedures based on simple arithmetic ratios of the proportions infected dose-by-dose. Top panels (**a**–**c**) assume distributions of susceptibility factors with the same mean, less than one (0.2), and different variances (**a**, 0 (homogeneous); **b**, 0.0533; **c**, 0.160 (all-or-nothing)). Bottom panels (**d**, **e**) use the susceptibility distributions estimated in King et al.^1^ (**d**, mean 1 and variance 0 for noncarriers, and mean 6.92 and variance 143 for carriers; **e**, mean 1 and variance 2.78 for noncarriers, and mean 1.49 and variance 10.9 for carriers). The threshold separating increased from reduced mean susceptibility is marked by dotted black lines.

We next address a comment by Ant et al.^4^ that our results contradict other studies. There is no contradiction in the sense that our methods are more general and coincide with those employed by others, when specific assumptions are made. To verify this, suppose that an individual is challenged with *d* infectious units (pathogen dose) and that each unit has a probability *p* of causing infection (host susceptibility). Assuming that the number of units effectively causing infection is Poisson distributed with mean *pd*, the probability of infection is 1−*e*^−*pd*^. Plotting this probability over a range of doses results in the dose–response curves represented in blue in Fig. 2. In a host population with homogeneous susceptibility, *p* can be estimated by challenging individuals with doses distributed over a suitable range and fitting the model to the resulting data. We can also relax the condition that the host population is homogeneous and assume that *p* is distributed according to some model^13^, in which case model fitting will give the distribution of host susceptibilities. Applying this procedure to published data, we concluded that *A. aegypti* mosquitoes in Brazil^1^ performed as homogeneously susceptible to dengue virus infection when challenged by intrathoracic injection with samples isolated from a local patient, whereas another set, with mosquitoes collected in Australia and infective blood drawn from patients in Vietnam^2^, supported gamma-distributed susceptibility under more natural blood-feeding challenges. Ant et al.^4^ criticise the inclusion of an intrathoracic injection dataset in our study on the grounds that it represents a substantial deviation from the natural infection route. In performing a comparative analysis between this and a blood-feeding dataset, however, we may be uncovering how variation builds into the system as the experimental procedure approaches natural conditions^3^.

We then extended the model to enable a distribution of *Wolbachia* effects to be inferred by simultaneously fitting carrier and noncarrier dose–response measurements^3^. Considering a homogeneous noncarrier population with per infectious unit probability of infection *p*, we write the susceptibility of a carrier individual as *xp*, where *x* is a susceptibility modifier due to *Wolbachia*. Individual variability in *Wolbachia* effects is accounted for by building a distribution into the dose–response model, which becomes $1 - {\int} {e^{ - xpd}q\left( x \right)dx}$, where *q*(*x*) is the distribution of susceptibility factors due to *Wolbachia*. In Fig. 2, we show several pairs of dose–response curves concurrently with the respective distributions *q*(*x*), and how different distributions of susceptibility affect the interpretation of differences in the response to infection among two groups, in this case mosquitoes with and without *Wolbachia*. The distribution of susceptibility factors due to *Wolbachia* is independent of dose; its mean is shown (solid black lines) below each set of dose–response curves, where it can be contrasted with simple dose-by-dose ratios of infected proportions^5–10^ (dashed black curves). It can be noted in the figure, that the actual mean susceptibility of *Wolbachia* carriers is often incorrectly estimated when using the standard single-dose approach, except in the very special case of *Wolbachia* conferring complete resistance to some individuals and none to others (Fig. 2c). The other extreme approach, which measures how much *Wolbachia* shifts the dose–response curve along the dose axis (in log scale)^2^, carries an implicit assumption that the symbiont affects all individuals equally (Fig. 2a). King et al.^3^ showed that neither of these two common approximations (heterogeneous all-or-nothing or homogeneous susceptibility of Wolbachia carriers) was supported by data. *Wolbachia* does not only contract or stretch dose–response curves along one axis or the other, but changes their shape, suggesting alternative distributions of susceptibility and therefore requiring more sophisticated analyses. This is illustrated in the bottom panels (Fig. 2d, e), which display point estimates of the distributions estimated in King et al.^3^ from dengue challenges in Brazil (Fig. 2d) and Vietnam (Fig. 2e). In these cases, *Wolbachia* was found to increase infection probability at low viral challenge doses (signalling increased mean susceptibility), while leading to shallower dose–response curves (increased variance) to meet reduction in infected proportions at high doses.

On the issue of uncertainty, Ant et al.^4^ also comment on the low numbers of mosquitoes positive for dengue virus at low challenge doses, which may result in low statistical power to support dose–response analyses. One of the benefits of fitting a model across a range of doses is precisely to increment statistical power by informing parameter estimation on multiple data points and extrapolating as appropriate^13^. Numbers infected are inevitably smaller at low challenges unless the experimental groups are incremented to compensate for the lower infection probabilities, but such optimal design strategy will not be adopted unless researchers are aware of its significance. It is entirely possible that large replication efforts might change the best estimates for the susceptibility distributions, and the analysis presented in King et al.^3^ is not dependent on a particular set of parameter values, or mean and variance effect of *Wolbachia*. Meanwhile, despite being based on low numbers and small effect sizes, the consistency of higher infected proportions among *Wolbachia* carriers at low challenge doses is noticeable. Ant et al.^4^ finalise their inspection into the Souto-Maior et al.^1^ dataset by noting a slight discrepancy between the raw data and the proportions infected shown in Fig. 1a of King et al.^3^. This is due to the exclusion of the earliest time point from the analysis (3 days post infection), both for reducing uncertainty and for uniformity with Ferguson et al.^2^. We have inadvertently bypassed this detail in the “Methods” of our original paper^3^. Ant et al.^4^ present similar arguments concerning our analysis of the Ferguson et al.^2^ dataset, defending dose-by-dose schemes and neglecting low doses, based on the rationale that our paper refutes.

One final point where we agree with Ant et al.^3^ is that “the need for consideration of virus in the saliva is also paramount”, which is why we used a dataset with such an approach^2^ to calibrate our model. This assessment of infectivity was conducted by straightforward procedures requiring only brief description which does not, however, diminish its importance. Integrating estimated *Wolbachia* effects on both susceptibility and infectivity, our transmission models predict reductions in dengue incidence in Vietnam, while in Brazil the analysis is less conclusive (King et al.^3^, Fig. 3).

In summary, we recognise that the original paper, especially the Abstract, did not adequately specify that an increase in mean susceptibility to dengue infection due to *Wolbachia* does not imply higher expected infection probability except at low viral challenge doses. We also did not place necessary caveats on the results noting the limitations of the prior datasets underlying our model (e.g., the existence of a small number of false positives). Nonetheless, we have shown here that our results are robust to a different threshold of data inclusion removing false positives, and that our model based on dose–response relationships reduces to more conventional approaches, when specific assumptions are made.

## Data Availability

All data supporting the findings of this study are available from the original publications Souto-Maior et al.^1^ and Ferguson et al.^2^.

## Code availability

Code used in this study is the same as in the original publication King et al.^3^ and can be obtained by contacting the authors.
